# Pain in adult myotonic dystrophy type 1: relation to function and gender

**DOI:** 10.1186/s12883-021-02124-9

**Published:** 2021-03-04

**Authors:** Gro Solbakken, Sissel Løseth, Anne Froholdt, Torunn D. Eikeland, Terje Nærland, Jan C. Frich, Espen Dietrichs, Kristin Ørstavik

**Affiliations:** 1 Institute of Clinical Medicine, University of Oslo, Oslo, Norway; 2 Department of Neurology, Rheumatology and Rehabilitation, Drammen Hospital, Vestre Viken Health Trust, Drammen, Norway; 3 Department of Clinical Medicine, The Arctic University of Norway, Tromsø, Norway; 4 Section of Clinical Neurophysiology, University Hospital of North Norway, Tromsø, Norway; 5 K.G. Jebsen Center for Neurodevelopmental Disorders, Institute of Clinical Medicine, University of Oslo, Oslo, Norway; 6 NevSom, Department of Rare Disorders, Oslo University Hospital, Oslo, Norway; 7 Institute of Health and Society, University of Oslo, Oslo, Norway; 8 Department of Neurology, Oslo University Hospital, Oslo, Norway

**Keywords:** Myotonic Dystrophy1, Pain, Gender, CTG size, Fatigue, Quality of life, motor function, BMI, FVC, psychological function, Autism quotient

## Abstract

**Background:**

Pain is prevalent in myotonic dystrophy 1 (DM1). This study investigated whether CTG repeat size, disease duration, BMI and motor and psychological function were related to pain in adult patients with DM1, and if there were gender differences regarding intensity and location of pain.

**Method:**

Cross-sectional design. Pain was investigated in 50 genetically confirmed DM1 patients by combining clinical assessment and self-reports of pain intensity and locations. Pain scoring results were related to CTG size, disease duration, muscle strength, walking capacity measured by 6-min walk test, activity of daily life by Katz ADL Index, respiratory function by Forced Vital Capacity and BMI. In addition, the degree of reported pain was related to Quality of life measured by WHOQOL-BREF; fatigue was measured by Fatigue severity scale; psychological functions were measured by Beck Depression Inventory, Beck Anxiety Inventory, IQ and Autism spectrum Quotient.

**Results:**

Pain was reported in 84% of the patients and was significantly correlated with CTG size (*r* = 0.28 *p* = 0.050), disease duration (*r* = 0.38 *p* = 0.007), quality of life (*r* = − 0.37 *p* = 0.009), fatigue (*r* = 0.33 *p* = 0.02) and forced vital capacity (*r* = − 0.51, *p* = 0.005). Significant gender differences, with higher scores for females, were documented. In male subjects the number of pain locations was significantly correlated with quality of life and the autism quotient. In females, pain intensity was significantly correlated with activity, respiratory function and BMI.

**Conclusions:**

Pain in DM1 was prevalent, with a strong association to lung function and other aspects of the disease. Significant gender differences were present for pain intensity and number of pain locations. How pain was related to other symptoms differed between male and female subjects. Our findings highlight the importance of assessments of pain in DM1 patients.

**Supplementary Information:**

The online version contains supplementary material available at 10.1186/s12883-021-02124-9.

## Introduction

Myotonic Dystrophy type 1 (DM1) is an inherited neuromuscular disease caused by an unstable CTG nucleotide repeat [1–3]. In addition to skeletal muscles, several organs and systems, such as the central and peripheral nervous system as well as endocrine organs and the eyes, may be affected. Cardiac and respiratory involvement is common [4, 5].

Pain in DM1 has mostly been studied by surveys [6–9]. Jensen et al. found that 60% of DM1 patients reported chronic pain, most frequently located in the lower back. Other areas included hands, legs, knees, ankles and feet, with a mean intensity of 4.5 measured on a numeric rating scale from 0 to 10 [6]. The authors also found a relationship between pain intensity and mobility, measured by Brief Pain Inventory (BPI) [6, 10]. Another study investigating pain in DM1 and fascioscapulohumeral muscular dystrophy (FSHD), concluded that pain intensity was related to psychological function [7]. A negative significant correlation between pain and general mental health measured by Short Form health Survey (SF-36) in DM1 was also documented by Smith et al. [9]. In another paper, Miró et al. found pain location to be important for the adjustment to pain, measured by BPI [8]. Objective testing and measures were lacking in this study [8]. Furthermore, large fiber neuropathy has been documented in the DM1 group, with a higher frequency in men than women, and with an association with BMI [11, 12] .

Gender has been shown to be related to several symptoms documented in patients with DM1. Men have more frequent cognitive impairment, cardiac and respiratory involvement and severe muscle disability and are more often socially isolated. Women have more frequent cataract, dysphagia, digestive tract dysfunction, incontinence, thyroid disorder and obesity [13]. Peric et al. documented a relationship between pain intensity over the last few weeks and female gender in DM2, though they did not observe the same association in DM1 [14]. Gender influencing pain in DM1 has not previously been reported.

In this cross-sectional study of adult patients with DM1, we investigated gender differences regarding intensity and location of pain, and whether the degree of CTG expansion, disease duration, motor and psychological function were related to pain.

## Participants and methods

### Recruitment and inclusion

Adult patients with DM1 from two different regions in Norway were invited to participate in a large cross-sectional, clinical study [15, 16]. The congenital and childhood forms of DM1 were not included, due to their different clinical symptoms [17]. The inclusion period was between 2012 and 2017. Patients were contacted through their respective hospitals, the National registry of neuromuscular diseases and the Norwegian patient organization. Fifty-five patients with a genetically verified diagnosis and a typical history of adult form of DM1, were invited to participate. Of these, 50 patients accepted.

All patients underwent a neurological examination with focus on motor function, and all the data included in this paper were collected during the outpatient visits.

### Disease measures: disease duration and CTG size

Disease duration was defined as time between onset of typical symptoms of DM1, which included myotonia, cataract, motor impairment or arrhythmia, and study enrollment [4]. Southern blot analysis for CTG size [1] was obtained from 49 patients at the time of inclusion.

### Pain measures

**Fig. 1:**
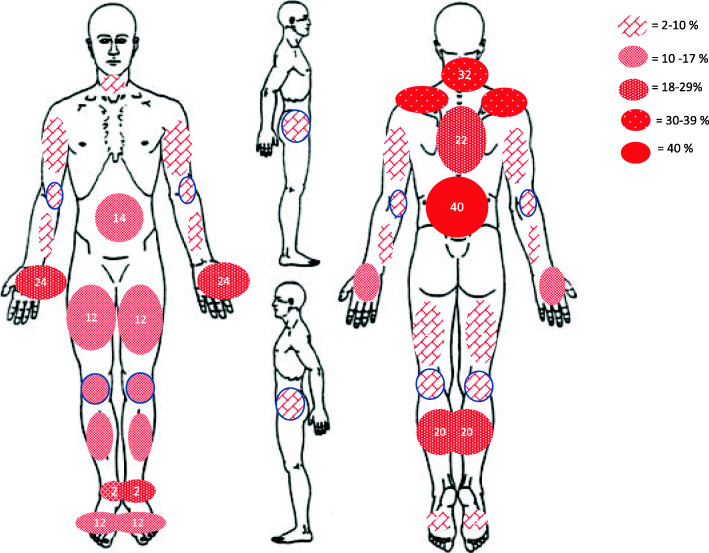
Pain-distribution (%), all participants

Patients were instructed to mark and score the intensity of chronic pain, which was defined as pain, excluding headache, that had been present for at least 3 months. Pain locations were identified using “pain drawings” (Fig. 1) and the number of pain sites was added up from these drawings [18]. Pain intensity was scored by the subjects as the experienced mean pain intensity based on the numeric rating scale (NRS 0–10): no pain = 0, mild pain = 1–3, moderate pain = 4–6, severe pain = 7–10 [18].

Descriptions of pain quality, which included aching, deep, burning, lancinating or electrical shock, were collected during history taking. Use of analgesics, physiotherapy or other pain management strategies, as well as perceived effect of these strategies, were documented.

### Functional measures and questionnaires

Muscle strength was assessed by the Medical Research Councils (MRC) manual muscle strength test (MMT) 0–5 [19]. The MRC MMT 0–5 has been criticized for its unequal categorical width, providing only ordinal data, and for low discrimination between categories when used in clinical practice [20, 21]. To counteract these limitations, we used the MRC 0–3 scale, which has been recoded from the MRC 0–5 scale according to Vanhoutte et al. [22]. The mean of muscle strength was used in all analyses and it composed of the muscle strength in distal extremities (comprising wrist extensors and dorsal flexors of the ankle), proximal extremities (comprising shoulder abductors, elbow flexors, elbow extensors, and hip flexors, knee flexors, knee extensors), and trunk (comprising the trunk flexors/abdominals and the back extensors). Due to previously reported symmetry, only one side was tested [23]. The disease specific Muscular impairment rating scale MIRS [24] was used to investigate the severity of the muscular impairment. MIRS is a 5-point scale where 1 is no muscular impairment, 2 is minimal signs, 3 distal weakness, 4 proximal weakness and 5 severe proximal weakness. The MIRS is reliable and has been validated, though Mathieu et al. advise caution when interpreting small samples due to low interrater reliability [24].

The six-minute walk test (6MWT) was conducted according to the American Thoracic Society guidelines [25]. However, the corridor track in the present study was 20 m long instead of 30 [26, 27]. Mean 6-min walking distance (6MWD) for healthy men between 20 and 50 years is 638 ± 44 m. Mean 6MWD for healthy women is 593 ± 57 m. These values are dependent on age and BMI in healthy populations [26]. Percent-predicted value for the 6MWD was calculated using reference equations as described by Enright et al.: men:1.140 m – (5.61 x BMI)- (6.94 x age), women: 1.017 m-(6.24 x BMI) – (5.83 x age) = [28].

The Katz Index of Independence in Activities of Daily Living was assessed by interview, to evaluate personal independence in activity of daily living [29, 30]. Forced Vital Capacity (FVC) values [31] and information on possible co-morbidities such as diabetes and thyroid dysfunction and symptomatic medication for myotonia were collected from the patients’ medical files. The fatigue severity scale (FSS) was used to measure general fatigue [32–35]. Scores > 5 on FSS are regarded as high levels of fatigue [32]. Height and weight were measured and body mass index (BMI) [36] was calculated.

### Cognitive measures and psychological questionnaires

General cognitive function (IQ) was assessed with the Wechsler Adult Intelligence Scale (WASI II) [37]. Symptoms of anxiety and depression were assessed with the Beck Anxiety Inventory (BAI) [38] and the Beck Depression Inventory (BDI) [39]. The Autism Spectrum Quotient (AQ) [40] was used for assessing the amount of ASD symptoms. And finally, for quality of life, the WHO quality of life-BREF (WHOQOL-BREF) questionnaire was utilized [41].

### Statistics

The SPSS 25 (IBM Corporation Armonk, NY, USA) was used for calculations. Normally distributed variables were presented with mean, standard deviation (SD) and range. Non-normally distributed variables were presented with median and range. Assessments of group differences were performed with students t-tests and Mann Whitney u test when appropriate. Effect sizes (Cohens d) were calculated using the online social science statistics service: http://www.soscistatistics.com/effectsize/Default3.aspx.

Correlations were performed with parametric and nonparametric tests when normal distributions were not present. *P*-values were set at two tailed = < 0.05, and Bonferroni corrections were used for adjustments of multiple comparisons for the question of gender differences regarding pain intensity measured by NRS and number of pain locations (NPL). Exact *p*-values were reported when between 0.05–0.001. All analyses of the pain measures and other disease characteristics are descriptive correlations, and not Bonferroni corrected due to their explorative nature. In order to control for group differences in CTG between men and women, a linear regression model with forced entry method was done for NRS, AQ, FVC and 6MWT. Assumptions for linear regression were met. We calculated 50 patients to be sufficient for 90% power to answer the questions of gender differences in pain.

### Guidelines

This study has been performed in accordance with the declaration of Helsinki. All methods and reporting were performed in accordance with the STROBE guideline.

## Results

### Participant characteristics

Fifty genetically verified adult DM1 patients were included. The patients had mild to severe reduction of muscle strength in trunk and distal extremities, while the proximal extremity muscle groups were mildly to moderately affected (Table 1). The CTG repeat size was distributed as follows in the 50 patients: 3 patients (6%) had a very small repeat size (50–100 CTG repeats), 6 patients (12%) had a small repeat size (101–200 CTG repeats); 24 patients (49%) had a medium repeat size (201–700 CTG repeats); and 16 patients (33%) had a large repeat size (> 700 CTG repeats); None of the patients were diagnosed with diabetes mellitus or thyroid dysfunction, or used medication for myotonia. Patient characteristics are summarized in Table 1.

**Table 1:** Characteristics of 50 patients with DM1

Characteristics	Mean, SD, (Range)	Men (n 24)	Women (n 26)	Difference between men and women/Cohens *d*
Age, years	40.1, SD: 12.6. (19–63)	37, SD: 13.8 (19–63)	43, SD: 10.7 (23–62)	ns
CTG kb N49	1.8, SD: 1.4 (0.230–5.4)	1.3, SD: 1.1 (0.270–4.7) n24	2.3, SD: 1.5 (0.230–5.4) n25	*P* = 0.008/0.8
Disease duration, years	19.0, SD: 10.0 (5–42)	16.4, SD: 9.6 (5–42)	21.5, SD: 9.7 (6–40)	ns
Mean strength of trunk muscles (MTT 0–3)	1.7, SD: 0.5 (1–2.6)	1.8, SD: 0.5 (1–2.6)	1.6, SD: 0.4 (1–2.3)	ns
Mean strength of distal extremity (MTT 0–3)	2.3, SD: 0.5 (1.5–3)	2.3, SD: 0.5 (1.5–3)	2.3, SD: 0.5 (1.5–3)	ns
Mean strength of proximal extremity (MTT 0–3)	2.6, SD: 0.3 (2–3)	2.7, SD: 0.3 (2–3)	2.6, SD: 0.3 (2–3)	ns
Fatigue (FSS) Questionnaire N48	4.8, SD: 1.3 (2–7)	4.5, SD: 1.6 (2–7) n23	4.9, SD: 1 (3–7) n25	Ns / 0.33
Walking test (6MWT) N45	382.4 SD:117.6 (123–615)	417.5, SD:103.4 (123–523) n23	348.8, SD:122.8 (140–615) n22	*p* = 0.049/0.60
Katz ADL Questionnaire	[6.0] (3–6)	[5.5] (3–6)	[6.0] (4–6)	ns
Autism quotient index (AQ) Questionnaire N47	17.0, SD: 6.1 (6–32)	19.4, SD: 5.4 (11–32) n21	15.0, SD: 6.1 (6–28) n 26	*p* = 0.014 / 0.75
IQ N41	92.0, SD: 14.2 (64–137)	92.5, SD:17 (64–137) n20	91.5, SD: 11.4 (71–114) n21	ns/ 0.06
Anxiety (BAI) Questionnaire N43	[4.0] (0–26)	[3.0] (0–26) n22	[4.5] (0–23) n 22	ns
Depression (BDI) Questionnaire N44	[8.0] (0–37)	[7.0] (0–19) n21	[8.0] (0–37) n 23	ns
Respiration spirometry (FVC%) N 29	71.7, SD: 18.8 (25–103)	76.4, SD: 14.0 (55–103) n13	67.9, SD: 21.6 (25–99) n16	ns/ 0.77
Quality of life (WHO QOL BREF) Questionnaire	89.7, SD: 13.3 (59–115)	91.3, SD: 12.4 (71–115) n24	88.2, SD:14.1 (59–114) n26	ns/ 0.23
BMI	26.6, SD: 6.6 (15–53)	26.3, SD: 5.5 (15–34)	26.9, SD: 7.5 (17–53)	ns/0.09

Mean, [Median] SD, (min/max) are presented. Exact p-levels are given for differences < 0.05 between men and women and the Cohens d effect size is reported

*DM1* Myotonic Dystrophy type 1, *CTG* Cytosine, Thymine, Guanine, *MMT* Manual Muscle strength test, *FSS* Fatigue Severity Scale, *6MWT* Six-minute walk test, *Katz ADL* Assessment of Activities of Daily Living, *IQ* Intelligence quotient, *BAI* Becks Anxiety Inventory, *BDI* Becks Depression Inventory, *FVC* Forced Vital Capacity, *WHOQOL BREF* World health Organization Quality of Life Assessment, *BMI* Body mass index

Percent-predicted 6MWD (n45): 56.78%, SD: 16.1, range:17.76–101.64, median 58.97.

Women (n23): mean 57.23, SD: 18.77, range: 22.11–101.64, median 51.29.

Men (n22): mean 56.30. SD: 13.15, range: 17.76–75.60, median: 60.81.

Not all participants completed all questionnaires. When N is below 50, exact number is reported. Five patients were not able to perform the 6MWT because of the need for a wheel chair. Some did not complete cognitive assessments and the psychological questionnaires due to personal reasons, mostly lack of time. With regard to questionnaires, the lowest number of completed questionnaires was 83% (BAI). In addition, 81% of patients completed the IQ assessment. One patient did not have a new analysis of CTG repeats, and was consequently excluded from analyses which included this measure. All patients completed the pain measures. Details of muscle impairment severity are summarized in Table 2.

**Table 2:** MIRS distribution

MIRS	MIRS mean	MIRS 1	MIRS 2	MIRS 3	MIRS 4	MIRS 5
N 50	3.1, SD:1.1(1–5)	4%	30%	24%	34%	8%
Men n 24	3, SD:1.1(1–5)	8.3% (n2)	29.2% (n7)	25% (n6)	29.2% (n7)	8.3% (n2)
Women n26	3.2, SD: 1 (2–5)	0	30.8% (n8)	23% (n6)	38.5% (n10)	7.7% (n2)

Mean, SD, and range are reported for the total MIRS in the whole group and in men and women. Distribution to the different MIRS categories, is presented as number and percentage of patients for the whole sample and for men and women

*MIRS* Muscular impairment rating scale

The functions measured did not differ between male and female patients. Neither did BMI. However, some Cohens d effect sizes are > 0.5, which may indicate group differences for 6MWT, FVC and AQ in a larger group. A possible contribution of CTG size compared to gender for FVC, AQ and 6MWT was investigated using linear regression models including gender and CTG as independent variables, see Table 1 Additional file 1. The models show that CTG had a stronger contribution than gender to FVC and 6MWT, whilst for AQ, gender had a stronger contribution compared to CTG (R: 0.34, *p* = 0.066, Beta CTG = -0.068, *p* = 0.67. Beta Gender = 0.31, *p* = 0.053).

### Pain

#### Frequency, intensity and locations

Chronic pain was reported in 84% of the patients, and 32% of these reported severe pain (NRS 7–10). Mean pain intensity was moderate (NRS = 4.6), and the mean number of pain locations (NPL) was 3.2. For gender differences, see Table 3. Pain locations were widespread and symmetrically distributed. The most frequently reported pain locations were the lumbar and cervical parts of the spine and the palmar sides of the hands (Fig. 1).

**Table 3:** Mean pain intensity and number of pain locations in the whole group and in men and women

Pain measures	Whole group N50	Men (n24)	Women (n26)	Difference between men and women
Mean NRS	4.6, SD: 2.7 (0–9) [5]	3.2, SD: 2.7 (0–8)	5.8, SD: 2.0 (0–9)	asympt. *p* = 0.001
Mean NPL	3.2, SD: 2.4 (0–8) [3]	2.3, SD: 2.3 (0–6)	4.0, SD: 2.2 (0–8)	asympt. *p* = 0.01

Mean, Standard deviation: (SD) and (range) are reported. Pain intensity and number of pain locations are both sig. Different for men and women. Median:[]. Bonferroni correction *p* = 0.01

#### Pain qualities

Different pain qualities were described. Most of the patients reporting pain described it as aching or deep or both (62%). 22% reported a burning or lancinating pain quality in their feet, distal part of their legs and hands.

#### Analgesics, physiotherapy and other pain intervention

Of the 42 patients reporting pain, 16 (38%) used analgesic. The following medication was used: Paracetamol was used by 11 patients (26%), four used opioids (9%), Gabapentin was used by three persons (7%), cannabinoids were used by three persons (7%), antidepressants by one person (2%). Five (12%) patients were on more than one type of analgesic.

Seventeen (40%) of the patients reporting pain used physiotherapy consisting of exercise, musculoskeletal mobilization and massage. Acupuncture and manipulation were also used as symptomatic treatment. Of these, 6 patients (14%) combined analgesics and physiotherapy.

All patients experienced some pain relief, while 5 (12%) reported very good pain relief.

#### Pain correlated to CTG size, age, disease duration and somatic and psychological symptoms

Pain intensity was significantly correlated to CTG size (*r* = 0.28, *p* = 0.050), disease duration (*r* = 0.38, *p* = 0.007), quality of life (*r* = − 37, *p* = 0.009) and fatigue (*r* = 0.33, *p* = 0.02). The number of pain locations was correlated to disease duration (*r* = 36, *p* = 0.01) and quality of life (*r* = − 0.33, *p* = 0.01). No significant correlation between the pain measures and age, anxiety, depression, the autism quotient, IQ, muscle strength, MIRS, 6MWT, Katz ADL or BMI were found. The only significant correlation between pain and function measures was between NRS and FVC which were negatively correlated (*r* = − 0.51, *p* = 0.005).

### Pain differences in men and women

#### Pain frequency

**Fig. 2:**
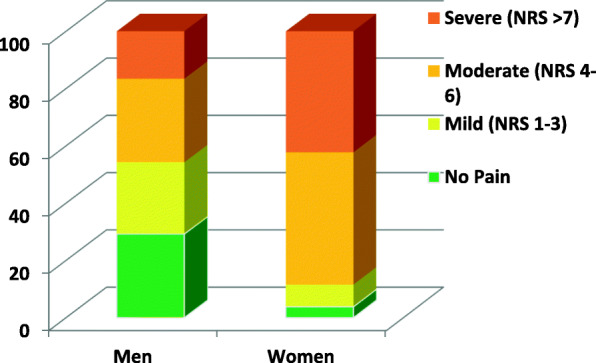
Distribution of pain intensity in men and women

Chronic pain was reported in 71% of the men and 96% of the women. Moderate pain was present in 46% of women and 25% of men, and the percentage of women reporting severe pain was more than twice that in men (42% vs. 20, see Fig. 2).

The distribution of pain locations differed between men and women. However, only pain in the back was significantly more frequent in women. 88% of the women reported back pain compared to 50% of the men (Fisher’s exact test *p* = 0.005).

#### Pain intensity and number of pain locations

NRS, as well as NPL were significantly different in men and women (Table 3).

In order to control for the group difference between gender in CTG size, one linear regression was conducted with NRS as a dependent variable. CTG size and gender were independent variables: *R* = 0.45, *p* = 0.003, only gender had an independent contribution: β: 0.39, *p* = 0.008.

#### Correlations between pain, CTG size, disease duration and symptoms in men and women

The correlation between NRS and disease duration was significant, but only in the female group (rho 0.41, *p* = 0.40). Furthermore, pain was related to different symptoms and findings in men compared to women, see Table 4.

**Table 4:** Correlations between pain and measures of function and quality of life in men and women

Measures	Men		Women	
	NRS /Pain Intensity	Number of pain locations	NRS/Pain Intensity	Number of pain locations
Quality of life / WHO QOL-BREF		rho = − 0.51 *p* = 0.012	ns	ns
Autism spectrum /AQ	ns	rho = 0.47 *p* = 0.03	ns	ns
Respiration / FVC	ns	ns	rho = −0.78 *p* = 0.0003	ns
ADL Katz	ns	ns	rho = −0.39 *p* = 0.048	ns
BMI	ns	ns	rho = 0.48 *p* = 0.016	ns

Exact *p*-levels are reported when < = 0.05

*ns* not significant = *p* > 0.05

Rho for Spearman’s correlation

In male subjects, NPL was significantly correlated to quality of life and to measures of socio-cognitive function (AQ). In female subjects, NRS was related to motor function measures, Katz ADL, FVC and to BMI. There was no significant association between pain and MMT.

## Discussion

In this study we found a high frequency of chronic pain with a mean value of moderate intensity in adult patients with DM1. Furthermore, pain was widespread and symmetrical, with 3.2 as the mean number of pain locations. In our sample of subjects, only NRS was significantly related to size of CTG repeats, FVC and fatigue. However, both NRS and the NPL were significantly correlated to disease duration and quality of life.

A novel finding was that there were gender differences for pain in DM1 patients. Pain was more frequent, had a greater intensity and was more widespread in female compared to male subjects. How pain is correlated to other symptoms also differs. In men, pain was correlated to the autism quotient (AQ), and quality of life (WHOQOL-BREF). In women, pain was related to disease duration, activity (Katz ADL), respiratory function (FVC) and BMI.

### Frequency, intensity and location

Most (84%) of the patients reported chronic pain, and 35% reported severe pain intensity (7–10 on NRS). This is consistent with findings from previous pain studies of DM1 where frequencies between 60 and 88% are described [6, 9, 14]. This frequency is higher than that which has been reported in general in Scandinavian populations. In Norway, the prevalence of chronic pain is reported to be 24% [42]. A review from Sweden and Denmark documented a prevalence of 16 and 18% respectively in non-cancer populations [43]. DM1 affects many organs and most patients can be characterized as multimorbid. Nociceptive pain may likely be present and caused by DM1 myopathy, where muscle tissue over time is replaced by fat infiltration, which leads to loss of function and strength [16, 44–46]. An additional cause may be neuropathic pain, since polyneuropathy is documented in DM1 groups without comorbidity like diabetes or thyroid dysfunction [12]. The quality descriptions of pain may differ in neuropathic compared to nociceptive pain. Pain quality may therefore, to a certain extent, differentiate causes of pain. 22% of the patients with pain in this study reported burning or lancinating pain, possibly indicating neuropathic pain.

The high number of pain locations documented in this study possibly reflects the distribution of muscular affection in DM1 as described in the literature [15, 23]. The most frequent pain locations reported by our patients were the lower back, the neck and the ventral side of the hands. Chronic low back pain is frequent in several populations [47]. Our findings indicate that chronic low back pain is even more frequent among adult DM1 patients than in mixed populations, an observation which was also documented in a study by Miro and co-authors [8, 47]. This might be caused by the trunk muscle impairments in DM1, which also includes severe atrophy of the lower erector spinae muscles [15, 16, 44, 45, 48]. This atrophy may not only cause loss of strength, but also impair co-contraction and muscle recruitment, and may lead to decreased stability of the trunk. Trunk stability is a prerequisite for mobility and for protecting the spine and extremities against injuries and possible pain [49]. In DM1, low back pain may be related to mechanical impairment and thereby nociceptive pain mechanisms such as increased weight on joints and the remaining skeletal muscle tissue.

In addition, weakness of the neck muscles, leading to inability to lift the head from a supine position, is a core clinical feature in DM1 [4]. Neck muscle atrophy may lead to problems with stabilizing the head and neck, thereby causing pain. Hand pain could be explained by the well-known myopathy in distal extremities in DM1 [4]. Indeed, in one case report, the patients’ presenting symptom of DM1 was pain in the hands [50].

### Pain, DM1 duration and CTG

Pain intensity was related to CTG size and disease duration. DM1 is a progressive disorder, and the severity of the disease depends to a high degree on the number of CTG repeats, which probably explains this finding [51]. Peric et al. also found that pain intensity was related to disease duration [14]. During progression of DM1, CTG levels increase, several organs and tissues may become affected, muscle strength decreases and motor and respiratory impairments increase [4]. As muscle strength decreases, activities of daily life become more challenging and possibly demand muscle activity closer to the maximal strength and endurance of the individual patients. Jensen et al. investigated pain intensity in groups with different mobility limitations. They found that pain intensity was significantly higher in DM1 patients with the most impaired mobility, in need of assistive devises for mobility, such as canes, a wheelchair or another person [6]. Pain in another neuromuscular disorder, congenital myopathy, is may be triggered by ADL activities [52]. Furthermore, because muscle work is dependent upon lung function, respiratory deficit may impair motor function even more. Also, in a study of DM1 mice, peripheral nerve affection was found to be related to CTG size [53]. Neuropathy may influence muscle function, both strength, endurance and timing may become impaired [54]. It is therefore possible that the impact DM1 progression and high levels of CTG repeats have on different organs, may lead to pain.

### Pain and anxiety, depression, fatigue and QoL

In contrast to the findings in previous studies on neuromuscular disorders, including DM1, we found no significant correlations between pain measures and anxiety or depression measured by BAI and BDI. This could be due to differences in study design, patient populations and measures [6, 7].

However, several findings in our study imply that pain has an impact on how patients feel and think. Both quality of life and the degree of fatigue were related to pain. Our documentation of an association between pain and fatigue is in line with studies in other patient populations [55]. Fatigue is also related to disease duration in DM1 [9, 56]. In addition, pain is shown to be predictive of fatigue in a longitudinal study on DM1 [57]. The relationship between pain and quality of life in DM1 is in line with findings in other neuromuscular disorders [8, 14, 58].

### Pain and gender

Gender differences in pain are well documented in general populations, with women being more frequently affected [42, 59]. We now document the same phenomenon in DM1. Female DM1 patients report a higher intensity and number of pain locations than male patients. The frequency of pain located in the lower back is also significantly higher in DM1 women. This may be related to the DM1 myopathy leading to lower muscle strength in women compared to men [16, 51]. Others have pointed out that the myopathy in DM1 mimics what is seen in sarcopenia [60]. In the elderly, a certain loss of muscle mass (sarcopenia) is related to chronic low back pain [61]. However, an association between chronic low back pain and the cross-sectional area of muscles is not clearly documented in healthy populations, whereas an association between pain and disability has been [62].

Interestingly, BMI did not differ in men and women. However, BMI was only related to pain in women. That pain is related to BMI is well documented in general populations, and mechanical impairments, as well as inflammation caused by the presence of adipose tissue are suggested as possible mechanisms for this [63]. In DM1, muscles become atrophied and replaced by fat infiltration, and muscle size is significantly smaller in female as compared to male patients [16]. One could speculate that inflammatory mechanisms, as well as mechanical causes may play a role in explaining the gender differences in DM1 patients.

The relation between pain and activity measured by Katz ADL seen in women might be explained by the lower level of muscle strength in DM1 women [51]. A relationship between pain and the most affected muscular regions is reported in FSHD [58]. However, we did not find an association between muscle strength and pain. This may be caused by the MMT measures not being sensitive enough for detecting smaller changes in muscle strength. Another explanation may be that DM1 women report higher pain intensity than DM1 men. Pain intensity > 4 on the Visual Analogue Scale is, to a lesser degree, related to activity [64]. The Katz ADL is composed of physical activity involving flexion, rotation and extension of the spine as well as gross and fine motor movements, which may be avoided to prevent or minimize pain.

We find lower levels of FVC in women than men, and a significant correlation between pain and FVC in women. This could be caused by women having a more restrictive respiratory pattern compared to men*.* The low FVC seen in women may be influenced by DM1 myopathy in the trunk muscles [16, 51]. Trunk muscles are important in respiration, and the rectus abdominus especially for forced expiration [16, 65]. Severe impairment of the cranial abdominal rectus has been documented in DM1 by our study group, as well as others [16, 44, 45]. FVC may thus be a predictor of a myopathic and impaired rectus muscles function, leading to pain. The relation between lung function and pain may also be caused by hypoventilation. Hypoventilation increases the probability of fatigue and reduced muscle endurance [66, 67].

The association between men’s pain and quality of life has previously been reported in a study on patients with chronic pain [68]. Given the same degree of chronic pain in male and female patients, that study also reported a significantly lower quality of life in men than in women [68]. This relationship may therefore be a general gender difference and not specific for DM1.

For the first time, a relation between symptoms of autism spectrum disease (ASD) and pain in DM1 is investigated. In previous pain studies on individuals diagnosed with autism, both hypo- and hypersensitivity are reported [69, 70]. Whether the correlation between pain and ASD symptoms identified in the current study are due to a common cause, or a result of abnormal self-reporting in individuals with high rates of ASD symptoms, is not known. It is possible that male patients become more attentive to their pain to the degree that it affects or impairs their social communicative function. However, the relationship observed may also reflect a particular disease trajectory in men where ASD symptoms co-occur with pain. Social communicative function such as ASD has been shown to be related to both neurophysiological and structural CNS abnormalities in DM1 [71, 72]. Another study on symptoms in DM1 found higher levels of CNS symptoms in male patients compared to female [13]. This might be in line with the latter explanation, but the relation between pain and social communicative function needs to be further explored in DM1.

Men and women differed significantly in CTG triplet size, and this could contribute to the gender differences in reported pain. However, in the regression model including CTG size and gender, only gender had a significant contribution. This strengthens the conclusion that there is a gender difference regarding pain in DM1, and that we as clinicians have to approach this symptom differently dependent on the sex of the patient.

### Strength and limitations

The main strength of our study is that we combine subjective and objective measures, in a well-defined patient group with genetic verification of the diagnosis for all participants. Another strength is that the clinical examination and measurements of the CTG size were both performed at the time of the pain assessments. Furthermore, patients were included in a broad assessment study and therefore probably not biased towards participating in a pain study only. This may have strengthened the study’s external validity to the general DM1 adult population. In addition, for the main question of gender differences, the sample size is large enough to make conclusions. A limitation is the cross-sectional design which does not allow for conclusions regarding which directions the relations go. Furthermore, caution must be made for some of the conclusions, given the explorative nature of some of the questions. And finally, since pain is subjectively measured by NRS and NPL, we cannot exclude gender differences in reporting style. We did not use a validated pain questionnaire like Brief Pain Inventory. This should be done in future studies investigating gender differences and pain in DM1. However, both the pain drawing and our measurement of pain intensity are the same as were used in BPI. The difference in CTG expansion size between men and women in our population represents a bias in our sample. Why the included women have more CTG repeats than men is not known, but may be due to the fact that mortality is higher in more severely affected DM1 men compared to women [13]. Another reason may be that DM1 men are more isolated and might therefore lack the personal support needed to respond to such an invitation [13].

## Conclusion

Pain in adult forms of DM1 is frequent and widespread. Mean pain intensity is moderate. Furthermore, pain is related to respiration, disease duration, quality of life, fatigue and CTG size. Pain in DM1 is influenced by gender and significant gender differences are present for pain intensity and number of pain locations. How pain is related to function is also different between men and women. In women, pain seems to be primarily related to respiration, BMI and motor function, while in men pain is more associated with psychological functioning.

Our findings highlight the importance of assessments of pain in DM1 patients, and associated symptoms. These gender-dependent relations between pain and function are important, and should be investigated in future research.

## Supplementary Information

**Additional file 1: Table 1.** Regression models controlling for CTG size and gender.

## Data Availability

The datasets generated and/or analysed during the current study are not publicly available due to the consent form used, some limitation of data sharing may apply, but are available from the corresponding author on reasonable request.
