# Supplementary material for: Pain in adult myotonic dystrophy type 1: relation to function and gender
Source: BMC Neurol. 2021 Mar 4;21:101. doi: 10.1186/s12883-021-02124-9 (PMC7931522; doi:10.1186/s12883-021-02124-9)
Supplement: Supplementary file 1 — Additional file 1: Table 1. Regression models controlling for CTG size and gender. [file 12883_2021_2124_MOESM1_ESM.docx]

**Additional file 1**

Table 1. Regression models controlling for CTG size and gender.

|  | Dependent: 6MWT | Dependent: FVC | Dependent: AQ |
| --- | --- | --- | --- |
|  | Model: R=0.61, **p=<0.0001** | Model: R= 0.56, **p=0.007** | Model: R= 0.34, p=0.066 |
| CTG | **Beta= -0.559,** **p=<0.0001** | **Beta=-0.560, p=0.004** | Beta=-0.068, p=0.666 |
| Gender | Beta=-0-114, p=0.395 | Beta=-0.007, p=0.967 | **Beta=-0.311, p=0.053** |
|  |  |  |  |
|  |  |  |  |
|  |  |  |  |

6-minute walk test (6MWT). Forced vital capacity (FVC). Autism Quotient (AQ). Cytosine Thymine Guanine trinucleotide repeat (CTG).

In these regression models where gender and CTG are independent variables, CTG is the most contributing factor to 6MWT and FVC while gender is the most contributing factor to AQ.
